# Epigallocatechin-3-gallate rapidly remodels PAP85-120, SEM1(45-107), and SEM2(49-107) seminal amyloid fibrils

**DOI:** 10.1242/bio.010215

**Published:** 2015-08-28

**Authors:** Laura M. Castellano, Rebecca M. Hammond, Veronica M. Holmes, Drew Weissman, James Shorter

**Affiliations:** 1 Department of Biochemistry and Biophysics, Perelman School of Medicine at the University of Pennsylvania, Philadelphia, PA 19104, USA; 2 Pharmacology Graduate Group, Perelman School of Medicine at the University of Pennsylvania, Philadelphia, PA 19104, USA; 3 Department of Biology, Swarthmore College, Swarthmore, PA 19081, USA; 4 Division of Infectious Diseases, Department of Medicine, Perelman School of Medicine at the University of Pennsylvania, Philadelphia, PA 19104, USA

**Keywords:** EGCG, SEVI, PAP85-120, SEM1, SEM2, HIV infectivity

## Abstract

Semen harbors amyloid fibrils formed by proteolytic fragments of prostatic acid phosphatase (PAP248-286 and PAP85-120) and semenogelins (SEM1 and SEM2) that potently enhance HIV infectivity. Amyloid but not soluble forms of these peptides enhance HIV infection. Thus, agents that remodel these amyloid fibrils could prevent HIV transmission. Here, we confirm that the green tea polyphenol, epigallocatechin-3-gallate (EGCG), slowly remodels fibrils formed by PAP248-286 termed SEVI (semen derived enhancer of viral infection) and also exerts a direct anti-viral effect. We elucidate for the first time that EGCG remodels PAP85-120, SEM1(45-107), and SEM2(49-107) fibrils more rapidly than SEVI fibrils. We establish EGCG as the first small molecule that can remodel all four classes of seminal amyloid. The combined anti-amyloid and anti-viral properties of EGCG could have utility in preventing HIV transmission.

## INTRODUCTION

Human immunodeficiency virus (HIV), which causes acquired immunodeficiency syndrome (AIDS), remains one of the most pressing global health challenges. The global HIV/AIDS prevalence rate is ∼0.8% and the majority of infections are transmitted heterosexually (UNAIDS, 2011). Semen harbors amyloid fibrils that potently enhance HIV infectivity *in vitro* (Arnold et al., 2012; Münch et al., 2007; Roan et al., 2014, 2011; Usmani et al., 2014). Specifically, proteolytic fragments of prostatic acid phosphatase (PAP248-286 and PAP85-120), semenogelin 1 (SEM1), and semenogelin 2 (SEM2) form fibrils that boost infectivity by electrostatically facilitating viral attachment to target cells (Arnold et al., 2012; Münch et al., 2007; Roan et al., 2014, 2011; Usmani et al., 2014). This enhancement of infection can be as large as several orders of magnitude and is independent of viral genotype and coreceptor tropism as well as the virus producer and target cell type (Kim et al., 2010). Remarkably, the stimulatory effect of SEVI (semen derived enhancer of viral infection) fibrils is greatest at low virus concentration, similar to the conditions observed in mucosal transmission of HIV, where relatively few virions traverse the mucosal barrier and initiate infection (Roan et al., 2009). Devising a method to rapidly remodel seminal amyloid fibrils into species unable to promote HIV infection would provide a novel and urgently needed preventative, microbicidal strategy for reducing sexual transmission of HIV (Castellano and Shorter, 2012).

We sought small molecules that might remodel seminal amyloid, as seminal fluid contains various proteases that could threaten the integrity of protein-based agents (Lundquist, 2008). However, small molecules that disrupt the highly stable, self-templating amyloid form remain rare (Roberts and Shorter, 2008; Shorter, 2010; Wang et al., 2008). One notable exception is epigallocatechin-3-gallate (EGCG), the major catechin from green tea, which exerts a wide range of antioxidant, anti-cancer, anti-aging, and anti-viral effects, while also exhibiting cardioprotective and neuroprotective properties (Cabrera et al., 2006; Khurana et al., 2013; Nance and Shearer, 2003; Yang et al., 2002). Interestingly, EGCG can potently inhibit the amyloidogenesis of various polypeptides and can also disassemble a wide range of preformed amyloid fibrils (Andrich and Bieschke, 2015; Bieschke et al., 2010; Cao and Raleigh, 2012; Chandrashekaran et al., 2011; Ehrnhoefer et al., 2008; Ferreira et al., 2011; Meng et al., 2010; Palhano et al., 2013; Roberts et al., 2009). Moreover, EGCG has been shown to: inhibit formation of PAP248-286 fibrils termed SEVI (Semen derived Enhancer of Viral Infection) via interaction with charged side chains (Popovych et al., 2012); dose-dependently deconstruct preformed SEVI fibrils (Hauber et al., 2009); and reduce both SEVI- and semen-mediated enhancement of HIV infection (Hartjen et al., 2012; Hauber et al., 2009). Importantly, EGCG (0.4 mM) was found to have an inhibitory effect on 41 out of 47 individual semen samples with a median inhibition of infection of ∼70.6% (Hartjen et al., 2012).

Here, we investigated the effect of EGCG on other seminal amyloid conformers formed by PAP85-120, SEM1(45-107), and SEM2(49-107) (Arnold et al., 2012; Roan et al., 2011). PAP85-120 is naturally found in human seminal fluid (Arnold et al., 2012), whereas SEM1(45-107) and SEM2(49-107) were initially suspected to be present in seminal fluid (Roan et al., 2011), but subsequent studies suggest that shorter peptides, e.g. SEM1(86-107), are naturally more abundant and also promote HIV infection (Roan et al., 2014). We found that EGCG rapidly remodels PAP85-120, SEM1(45-107), and SEM2(49-107) fibrils, and this remodeling occurs more rapidly than EGCG-driven remodeling of SEVI fibrils. Our findings establish EGCG as the first small molecule shown to remodel all four classes of seminal amyloid.

## RESULTS

### EGCG slowly remodels SEVI fibrils

**Fig. 1.**
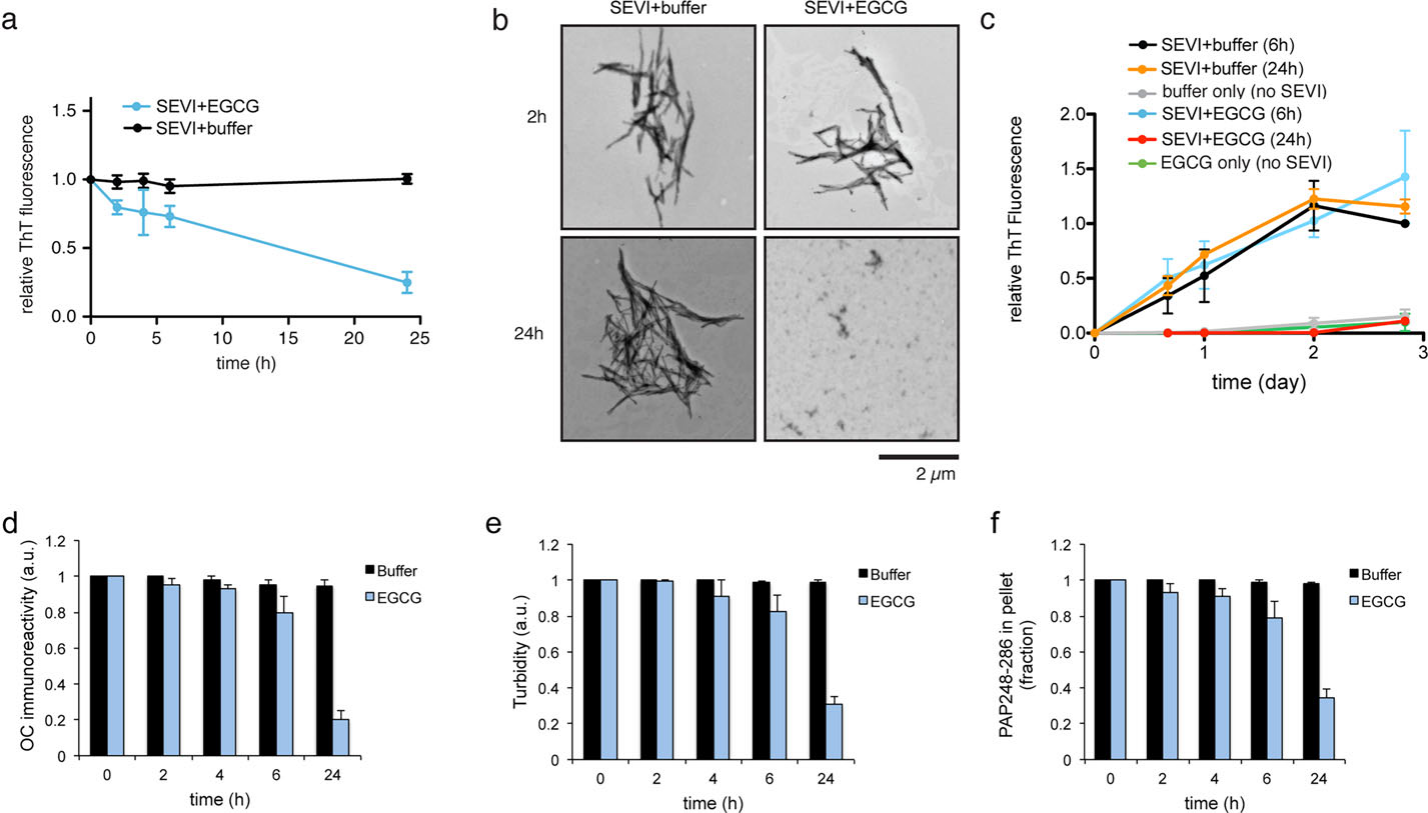
**EGCG slowly remodels SEVI fibrils into non-amyloid structures.** (A) Preformed SEVI fibrils (20 µM) were incubated with buffer or EGCG (200 µM) for 0–24 h. Fibril integrity was assessed by ThT fluorescence. Values represent means±s.e.m. (*n*=4). (B) Transmission electron micrographs of SEVI fibrils incubated with buffer or EGCG for 2 h or 24 h. Scale bar: 2 µm. (C) SEVI fibrils (20 µM) were incubated with buffer for 6 h or 24 h or EGCG (200 µM) for 6 h or 24 h, and the resulting products were used to seed soluble PAP248-286 (1 mM, 0.1% fibril seed) fibrillization. Buffer conditions lacking fibril seed were included. Fibril assembly was monitored by ThT fluorescence. Values represent means±s.e.m. (*n*=4). (D-F) Preformed SEVI fibrils (20 µM) were incubated with buffer or EGCG (200 µM) for 0–24 h. Fibril integrity was assessed by anti-amyloid (OC) immunoreactivity (D), turbidity (E), or sedimentation analysis (F). Values represent means±s.e.m. (*n*=3).

The small molecule EGCG, a potent antioxidant and polyphenol found in green tea, has previously been shown to dose-dependently disassemble SEVI fibrils over 24–48 h (Hauber et al., 2009). We confirmed this gradual disassembly, as a drastic decrease in thioflavin-T (ThT) fluorescence intensity was not observed until SEVI fibrils were treated with a ten-fold excess of EGCG for 24 h (Fig. 1A). Transmission electron microscopy (TEM) verified that fibrils were still the predominant species present after a 2 h treatment with EGCG (Fig. 1B). Furthermore, we found that SEVI fibrils pre-treated with EGCG for 6 h could still effectively ‘seed’ the fibrillization of monomeric PAP248-286 (Fig. 1C). Thus, EGCG is unable to eliminate self-templating activity or remodel SEVI into a non-amyloid form on this timescale (Fig. 1C). After a longer 24 h treatment, however, a striking change in morphology was observed by TEM, where significantly smaller oligomeric structures were observed in place of fibrils (Fig. 1B). SEVI fibrils pre-treated with EGCG for 24 h could no longer seed the assembly of PAP248-286 (Fig. 1C). We confirmed slow remodeling of SEVI fibrils by EGCG after 24 h, but not at earlier times, using three separate measures: immunoreactivity to the anti-amyloid OC antibody (Kayed et al., 2007) (Fig. 1D), turbidity (Fig. 1E), and sedimentation analysis (Fig. 1F). Thus, we confirm previous observations that EGCG remodels SEVI fibrils (Hauber et al., 2009).

### EGCG rapidly remodels PAP85-120 fibrils

Next, we explored the effect of EGCG on other amyloid fibrils present in semen. Since a multitude of seminal amyloid fibrils have been discovered, it would be advantageous to develop agents that possess broad activity against a range of amyloid conformers to effectively antagonize amyloid-mediated HIV infectivity enhancement (Castellano and Shorter, 2012). Hence, we investigated whether EGCG could disrupt PAP85-120, SEM1(45-107), and SEM2(49-107) fibrils, which can also promote HIV infection *in vitro* (Arnold et al., 2012; Roan et al., 2011).

**Fig. 2.**
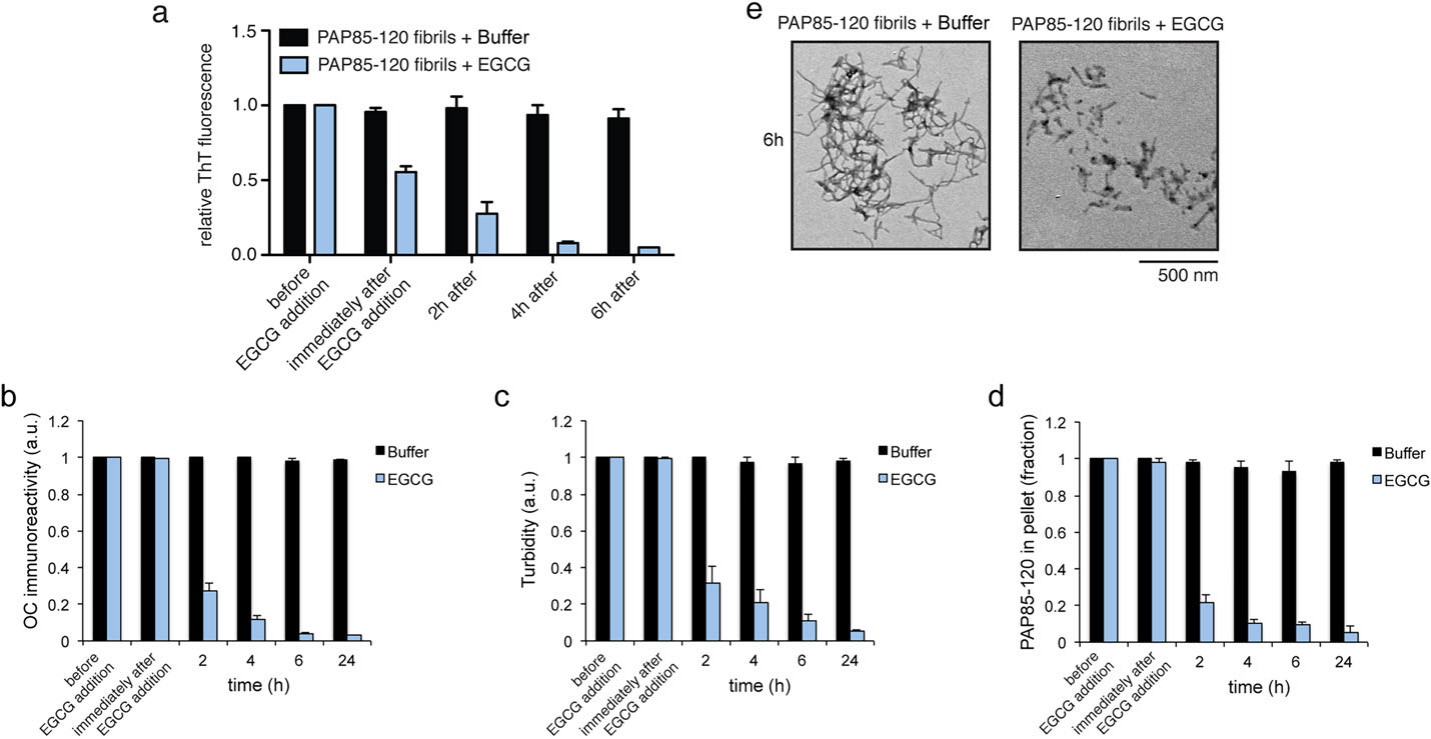
**EGCG rapidly remodels PAP85-120 amyloid fibrils.** (A-D) PAP85-120 fibrils (20 µM) were incubated with buffer or EGCG (200 µM) for 0−24 h. Fibril integrity was assessed by measuring: ThT fluorescence intensity (A), OC immunoreactivity (B), turbidity (C), or sedimentation analysis (D). Values represent means±s.e.m. (*n*=3). (E) Transmission electron micrographs of PAP85-120 fibrils incubated with buffer (untreated) or EGCG for 6 h. Scale bar: 500 nm.

Using a ten-fold excess of EGCG, we found that the ThT fluorescence intensity of PAP85-120 fibrils decreased to ∼55% of the initial value immediately after the addition of EGCG and decreased by ∼95% after 6 h (Fig. 2A). Several studies have shown that EGCG does not interfere with ThT fluorescence by some non-specific mechanism (Bieschke et al., 2010; Cao and Raleigh, 2012; Meng et al., 2010; Roberts et al., 2009). Thus, we attribute this rapid decay of ThT fluorescence to rapid fibril remodeling, which has also been observed with EGCG and amylin fibrils (Cao and Raleigh, 2012; Meng et al., 2010). However, using three other measures of fibril integrity we did not observe any alteration immediately after addition of EGCG, but remodeling was apparent after 2 h (Fig. 2B-D). Thus, ThT fluorescence might detect a very early event in PAP85-120 fibril remodeling by EGCG, such as remodeling of ThT-binding sites, or EGCG might interfere with ThT binding-associated fluorescence (Palhano et al., 2013). Nonetheless, all measures of fibril integrity indicated that the remodeling of PAP85-120 by EGCG occurred significantly more rapidly than EGCG-driven remodeling of SEVI fibrils (Fig. 2), which were largely intact after 6 h (Fig. 1A,D-F). Analysis of PAP85-120 fibrils treated with EGCG for 6 h by TEM showed predominately small oligomeric species, as well as a few short fibrils (Fig. 2E). Thus, EGCG rapidly remodels PAP85-120 fibrils.

### EGCG rapidly remodels SEM1(45-107) and SEM2(49-107) fibrils

**Fig. 3.**
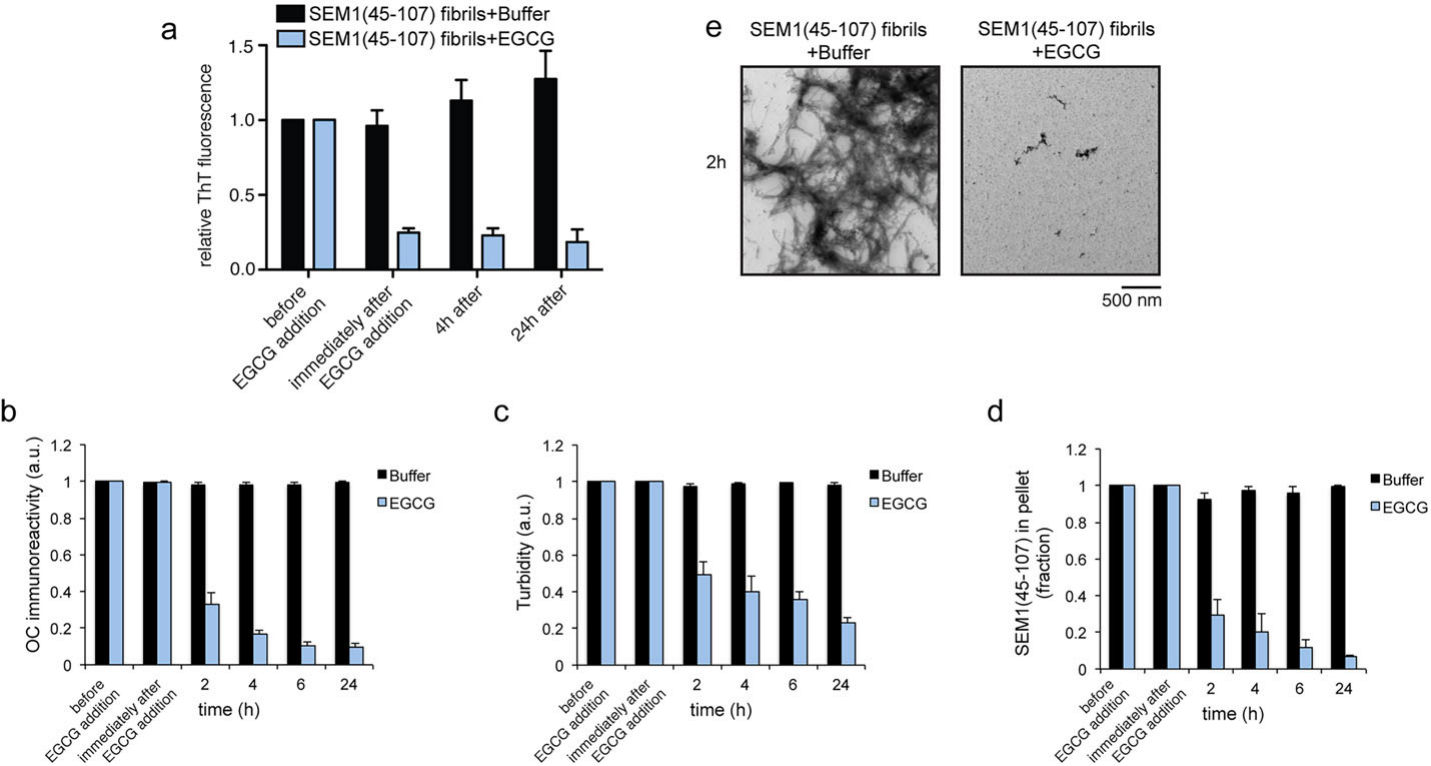
**EGCG rapidly remodels SEM1(45-107) fibrils.** (A) SEM1(45-107) fibrils (20 µM) were incubated with buffer or EGCG (200 µM) for 0–24 h. Fibril integrity was assessed by measuring: ThT fluorescence intensity (A), OC immunoreactivity (B), turbidity (C), or sedimentation analysis (D). Values represent means±s.e.m. (*n*=3). (E) Transmission electron micrographs of SEM1(45-107) fibrils incubated with buffer or EGCG for 2 h. Scale bar: 500 nm.

**Fig. 4.**
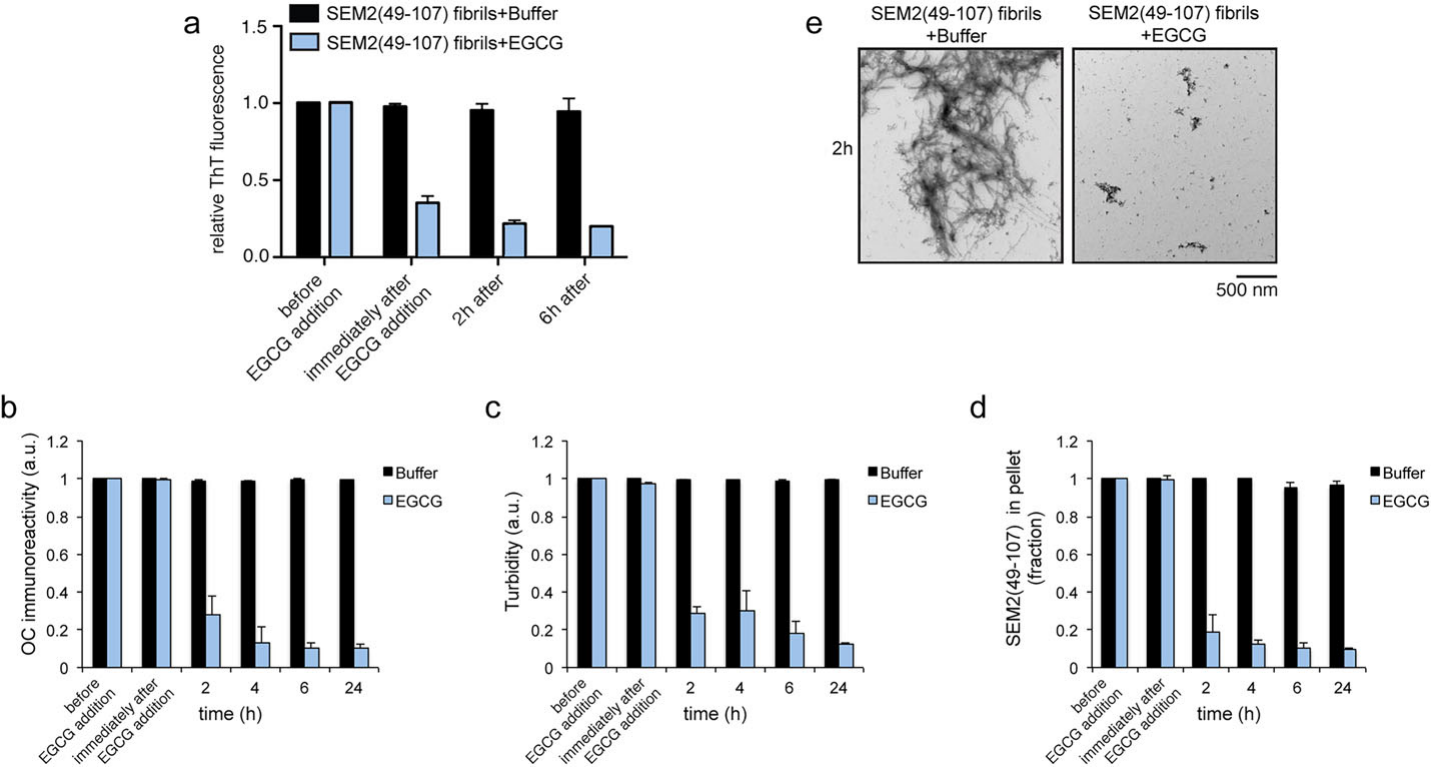
**EGCG rapidly remodels SEM2(49-107) fibrils.** (A) SEM2(49-107) fibrils (20 µM) were incubated with buffer or EGCG (200 µM) for 0–24 h. Fibril integrity was assessed by measuring: ThT fluorescence intensity (A), OC immunoreactivity (B), turbidity (C), or sedimentation analysis (D). Values represent means±s.e.m. (*n*=3). (E) Transmission electron micrographs of SEM2(49-107) fibrils incubated with buffer or EGCG for 2 h. Scale bar: 500 nm.

Next, we tested whether EGCG could also remodel SEM1(45-107) fibrils. The ThT fluorescence intensity decayed drastically to ∼25% of the initial value for SEM1(45-107) immediately following the addition of a ten-fold excess of EGCG (Fig. 3A). Only a minor additional decline in ThT intensity to ∼18% of the initial value was observed for SEM1(45-107) fibrils after 24 h of incubation with EGCG (Fig. 3A). OC immunoreactivity, turbidty, and sedimentation analysis also revealed that EGCG remodeled SEM1(45-107) fibrils, but as with PAP85-120 fibrils, remodeling assessed by these measures was only observed at 2 h or later (Fig. 3B-D). Examination by TEM revealed that EGCG-remodeled SEM1(45-107) products were very small oligomeric structures (Fig. 3E). Very similar observations were made with SEM2(49-107) fibrils, which were also rapidly remodeled by EGCG (Fig. 4A-E). These findings suggest that EGCG disrupts critical contacts that are required to maintain SEM1(45-107) and SEM2(49-107) fibrils.

### EGCG inhibits HIV infectivity in cell culture

**Fig. 5.**
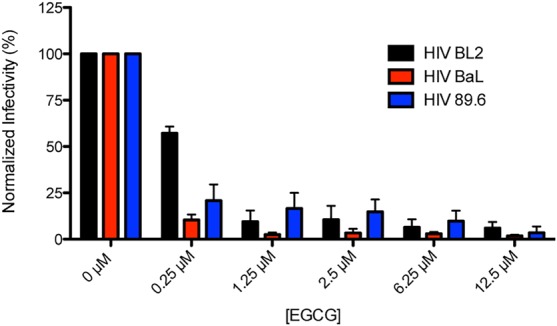
**EGCG inhibits HIV infectivity in cell culture.** TZM-bl cells were infected with three HIV-1 strains (BL2, BaL, and 89.6) in the presence of the indicated concentrations of EGCG (final concentration in cell culture). Infectivity was monitored by measuring luciferase activity in the cell cultures. Values represent means±s.e.m. (*n*=3).

EGCG is the first agent that has been found to disrupt the amyloid architecture of all four classes of seminal amyloids that have been identified (SEVI, PAP85-120, SEM1, and SEM2). Previous work reported that EGCG counteracts the viral infection enhancing activity of SEVI (Hauber et al., 2009). Thus, we next wanted to determine whether the products of PAP85-120, SEM1(45-107) and SEM2(49-107) fibril remodeling by EGCG also had a reduced capacity to boost HIV infectivity. Unfortunately, however, our analysis was confounded, since EGCG on its own exhibited a marked anti-viral effect against three different HIV strains (Fig. 5). At a concentration of only 0.25 µM EGCG, viral infectivity was reduced to ∼61%, ∼35%, and ∼11% of the control condition against the HIV-1 viral strains BL2, BaL, and 89.6, respectively (Fig. 5). When the EGCG concentration was increased to 1.25 µM or higher, the infectivity of all three strains was essentially abolished. In accord with previous studies (Bieschke et al., 2010; Ehrnhoefer et al., 2008; Hauber et al., 2009), none of the EGCG concentrations tested were toxic to cells as determined by 3-(4,5-dimethylthiazol-2-yl)-2,5-diphenyl tetrazolium bromide (MTT) assay (data not shown). The direct anti-HIV effect of EGCG has been previously described and is proposed to occur through a variety of mechanisms (Fassina et al., 2002; Kawai et al., 2003; Steinmann et al., 2013; Yamaguchi et al., 2002). This direct anti-viral property in combination with the ability of EGCG to disaggregate SEVI, PAP85-120, SEM1(45-107) and SEM2(49-107) seminal amyloids highlight the potential for the use of EGCG in a preventative HIV microbicide with dual mechanisms of action.

## DISCUSSION

Here, we show that in addition to remodeling SEVI fibrils (Hauber et al., 2009), EGCG can rapidly remodel PAP85-120, SEM1(45-107), and SEM2(49-107) fibrils, making it the first reported agent that can remodel all four classes of seminal amyloid identified to date. Indeed, this broad-spectrum activity distinguishes EGCG from the lysine- and arginine-specific molecular tweezer, CLR01 (Fokkens et al., 2005), which can remodel SEVI and PAP85-120 fibrils, but not SEM1(45-107) and SEM2(49-107) fibrils (data not shown) (Lump et al., 2015). Likewise, the amyloid-remodeling factor and AAA+ ATPase from yeast, Hsp104 (Shorter, 2008), and its potentiated variant, Hsp104^A503V^ (Jackrel et al., 2014), could remodel SEVI and PAP85-120 fibrils, but not SEM1(45-107) and SEM2(49-107) fibrils (data not shown) (Castellano et al., 2015). Thus, EGCG is unusual in its ability to remodel SEM1(45-107) and SEM2(49-107) fibrils.

EGCG remodeled SEVI fibrils into species that were unable to seed the assembly of soluble PAP248-286 (Fig. 1C). Interestingly, we have found that fibrils formed by PAP85-120, SEM1(45-107), and SEM2(49-107) exhibit poor seeding activity even before they were treated with EGCG (data not shown). Thus, we did not assess the seeding activity of EGCG-remodeled PAP85-120, SEM1(45-107), and SEM2(49-107). Poor seeding activity can be a feature of amyloids that form via downhill polymerization as opposed to nucleated conformational conversion, as is the case with transthyretin amyloid fibrils, which exhibit poor seeding activity (Hurshman et al., 2004; Lai et al., 1996; Westermark and Westermark, 2008).

PAP85-120, SEM1(45-107), and SEM2(49-107) fibrils were remodeled by EGCG more rapidly than SEVI fibrils, indicating that the cross-β contacts that maintain PAP85-120, SEM1(45-107), and SEM2(49-107) fibrils are more susceptible to disruption by EGCG. However, because we also observed complete inhibition of three HIV strains by micromolar concentrations of EGCG in our experimental paradigm, we were unable to investigate the infectivity-enhancing potential of the remodeled products. By contrast, Hauber et al. established conditions with Jurkat cells where millimolar concentrations of EGCG did not directly inhibit HIV infection (Hauber et al., 2009). In this context, EGCG inhibited the ability of SEVI fibrils to promote HIV infection (Hauber et al., 2009). However, in other experiments using the same TZM-bl cell line we employed, Hauber et al. do not appear to report the EGCG plus virus alone control (see figure S3 in Hauber et al., 2009). Based on our results (Fig. 5), we therefore suggest that the major inhibitory effect of EGCG on HIV infection of TZM-bl cells reflects a direct effect on the virus and not on SEVI fibrils.

EGCG has also been shown to inhibit the infection-enhancing properties of both SEVI and semen (Hartjen et al., 2012; Hauber et al., 2009). In a minority of individual semen samples, however, this enhancement was resistant to EGCG treatment, and reasons for this variability remain to be further elucidated. In this regard, it is interesting to note that EGCG can exhibit differential ability to remodel distinct cross-β structures, termed strains, formed by the yeast prion protein, Sup35 (Duennwald and Shorter, 2010; Roberts et al., 2009; Shorter, 2010). By analogy, it is plausible that seminal peptides might also be capable of assembling into EGCG-resistant amyloid polymorphs in a minority of individuals. It therefore becomes important to elucidate small molecule combinations that disrupt all seminal amyloid strains (Duennwald and Shorter, 2010; Roberts et al., 2009; Shorter, 2010).

Finally, EGCG exhibits pronounced antiretroviral effects in the absence of seminal amyloid and affects various steps in the HIV replication cycle. Specifically, EGCG inhibits cell entry by obstructing the attachment of viral gp120 to CD4T cells (Kawai et al., 2003; Nance et al., 2009), and viral replication through inhibition of Tat-induced LTR transactivation (Zhang et al., 2012). EGCG has also been proposed to function as an allosteric reverse transcriptase inhibitor (Li et al., 2011) and an integrase inhibitor (Jiang et al., 2010). The combined anti-amyloid and anti-viral effects of EGCG make it a promising polypharmacological candidate for use in a vaginal or anal microbicide with diverse modes of action. Moreover, the ability of EGCG to remodel SEM1(45-107) and SEM2(49-107) fibrils could complement existing amyloid-remodeling strategies based on CLR01 or Hsp104, which fail to remodel these fibrils (Castellano et al., 2015; Lump et al., 2015).

## MATERIALS AND METHODS

### Reagents

EGCG was obtained from Sigma-Aldrich and stock solutions of 10 mM were freshly prepared in aqueous buffer. The anti-amyloid antibody, OC (Kayed et al., 2007), was from Millipore.

### Peptides and amyloid formation

SEVI (PAP248-286), PAP85-120, SEM1(45-107), and SEM2(49-107) peptides were obtained from Keck Biotechnology Resource Laboratory (Yale University, New Haven, CT). Lyophilized peptides were reconstituted and assembled into fibrils as described (Arnold et al., 2012; Münch et al., 2007; Roan et al., 2011). Briefly, lyophilized PAP248-286 was dissolved in PBS to 1 mM, passaged through a 0.2 µm filter, and agitated at 37°C and 1400 rpm (Eppendorf Thermomixer) for ∼72 h. All subsequent SEVI fibrils were assembled by adding 1% preformed fibril seed to soluble PAP248-286 solutions and agitating at 37°C and 1400 rpm overnight. Lyophilized PAP85-120 was first dissolved in 1,1,1,3,3,3-hexafluoro-2-propanol (HFIP) to remove preformed aggregates and separated into 100 µl aliquots. HFIP was removed by drying in a speed vacuum for 30 min. The resulting film was dissolved in Gibco UltraPure water to 1 mM, passaged through a 0.2 µm filter, and solutions were agitated at 37°C and 1400 rpm for 24–48 h. Lyophilized SEM1(45-107) or SEM2(49-107) were dissolved in 0.123 M sodium phosphate buffer to 0.5 mM, passaged through a 0.2 µm filter, and agitated at 37°C and 1400 rpm for 7 days. Peptide concentrations were calculated using extinction coefficients at 280 nm.

### Fibril remodeling

Seminal amyloid fibrils (20 µM) were incubated with a ten-fold excess of EGCG (200 µM) at 37°C, and fibril integrity was assessed by monitoring Thioflavin-T (ThT) fluorescence intensity (excitation: 440 nm, 5 nm bandwidth; emission: 482 nm, 10 nm bandwidth) using a Tecan Safire^2^ microplate reader. Alternatively, the anti-amyloid OC antibody was used to detect amyloid conformers via ELISA as described except that the coating time was reduced to 30 min (Kayed et al., 2007, 2003). Turbidity was also used to assess fibril integrity by measuring absorbance at 635 nm (Woods et al., 2011). For sedimentation analysis, reactions were centrifuged at 16,100 ***g*** for 10 min at 25°C. The amount of peptide in the supernatant and pellet fractions was then determined using the Bradford assay as described (Palhano et al., 2013). For all experiments, low protein-binding plasticware was employed.

### Transmission electron microscopy (TEM)

For TEM analysis, samples were spotted on formvar carbon-coated grids (EM Sciences), stained with 2% uranyl acetate, and visualized using a Jeol-1010 transmission electron microscope.

### Cell culture and HIV infectivity experiments

We used TZM-bl cells to report on HIV infection. TZM-bl cells are a HeLa cell line derivative that expresses high levels of CD4 and CCR5 along with endogenously expressed CXCR4. TZM-bl cells contain HIV LTR-driven beta-galactosidase and luciferase reporter cassettes that are activated by HIV Tat expression (Finnegan et al., 2004). TZM-bl cells were maintained in DMEM medium supplemented with 10% fetal bovine serine and 1% L-glutamine. To dose the inhibition of HIV infectivity by EGCG, EGCG was freshly dissolved in aqueous buffer and dilutions of various concentrations were prepared. These EGCG solutions (50 µl) were added to 50 µl DMEM. Next, 82.5 µl of the resulting mixture was preincubated with 82.5 µl HIV (300 µl) at room temperature for 10 min. When the preincubation was complete, media was removed from the 96-well plate harboring the TZM-bl cells, and the EGCG/virus mixtures were immediately added in triplicate (50 μl per well) to 10^4^ TZM-bl cells seeded in collagen-coated 96-well microplates. After 3 h at 37°C, mixtures were removed and replaced with 200 µl of complete media. Luciferase activity was determined at 3 days post infection using a MLX Microtiter Plate Luminometer. HIV-1 strains used included BL2 (a primary isolate derived from the blood that uses CCR5; 65 infectious units; 0.45 ng p24), BaL (37.5 infectious units; 0.21 ng p24), and 89.6 (500 infectious units, 0.46 ng p24).
